# Primary central nervous system diffuse large B-cell lymphoma in fourth ventricle: Case report and literature review

**DOI:** 10.1097/MD.0000000000033286

**Published:** 2023-03-24

**Authors:** Jiahui Zhao, Cunyi Zou, Zongze Guo, Peng Cheng, Weicheng Lu

**Affiliations:** a Department of Neurosurgery, The First Hospital of China Medical University, Shenyang, Liaoning, China; b The First Hospital of China Medical University, Heping District, Shenyang, Liaoning, China.

**Keywords:** diagnosis, fourth ventricle, primary central nervous system lymphoma, treatment

## Abstract

Primary central nervous system lymphoma (PCNSL) is rare, especially lymphoma arising in the fourth ventricle. Only a few cases have been reported. We report a case of fourth ventricular lymphoma and review the relevant literature. Characterizing these cases can provide a basis for optimizing the diagnosis and management of fourth ventricle lymphoma.

**Patient concerns:**

A 48-year-old male with blurred vision, dizziness, staggering persisting for 2 months was admitted.

**Diagnosis:**

Preoperative magnetic resonance imaging revealed a space occupying lesion of the fourth ventricle. The patient presented with symptoms of hydrocephalus before surgery, such as memory loss and slurred speech. Pathological analysis following complete resection confirmed the lesion as PCNSL.

**Intervention:**

The patient underwent a midline posterior fossa craniotomy.

**Outcomes:**

The patient symptoms were relieved after surgery. Postoperative chemotherapy was administered with our regular follow-up. Follow-up 9 months after operation indicated a good prognosis.

**Lessons:**

According to the literature, biopsy surgery and subsequent chemotherapy are generally considered as the best treatment options for PCNSL. We believe that for the special location of the fourth ventricle, lymphomas in this site are suitable for the combination of complete resection and subsequent chemotherapy. This approach facilitates tumor resection and reduces possibility of obstructive hydrocephalus.

## 1. Introduction

Primary central nervous system lymphoma (PCNSL) is scarce nonHodgkin lymphoma (NHL) with especially poor prognosis. It accounts for about 1% of all intracranial tumors.^[1]^ Most of intracranial lymphoma is diffuse large B-cell lymphoma. Most of the lesions are supratentorial and periventricular, often involving deep structures such as corpus callosum and basal ganglion.^[2]^ Isolated intraventricular lymphoma is rare and only a few case reports.

The exact pathogenesis of PCNSL is not well understood, and there are several hypotheses as follows:

1. EB virus infection can make B cells in immunocompromised patients escape from T cell surveillance and proliferate excessively, enter the nervous system and lead to PCNSL.
2. Lymph node B cells are activated and transformed into tumor cells, which migrate to the central nervous system through the blood to form lymphomas.
3. Some undifferentiated pluripotent stem cells in cerebral blood vessels eventually differentiate into tumor cells, leading to the occurrence of PCNSL.
4. The occurrence of PCNSL may be related to the expression of apoptosis genes. Bcl-2 gene is often highly expressed in lymphoma patients and inhibits cell apoptosis. The Bax and Bcl-x genes were lowly expressed, prolonging the survival of lymphocytes.
5. PCNSL has unique genomic alterations, including the deletion of chromosome 6p21 harboring the human leukocyte antigen locus, recurrent 9p21 losses, and 9p24.1 copy number alterations and translocations that encode programmed PD-L1 and programmed PD-L2.
6. MYD88 and CD79b mutations are very common in the pathogenesis of central nervous system lymphoma.^[3]^

PCNSL usually occurs in immunodeficiency people (most commonly HIV) but is increasing reported in immunocompetent people.^[1]^ Here, we report a case of PCNSL isolated to the fourth ventricle and summarize 11 cases of fourth ventricle PCNSL. We attempt to raise awareness of this rare tumor and describe our treatment experience with it, aiming to explore some methods and rules of clinical treatment.

## 2. Case report

### 2.1. History and examination

A 48-year-old male patient was admitted to our department presenting with blurred vision, dizziness, staggering. These symptoms had persisted for 2 months, and aggravated recently. He has no history of hypertension, diabetes, coronary heart disease, and other family history of disease. A neurologic examination suggested decreased vision in both eyes and poor muscle strength of both lower limbs.

**Figure 1.**
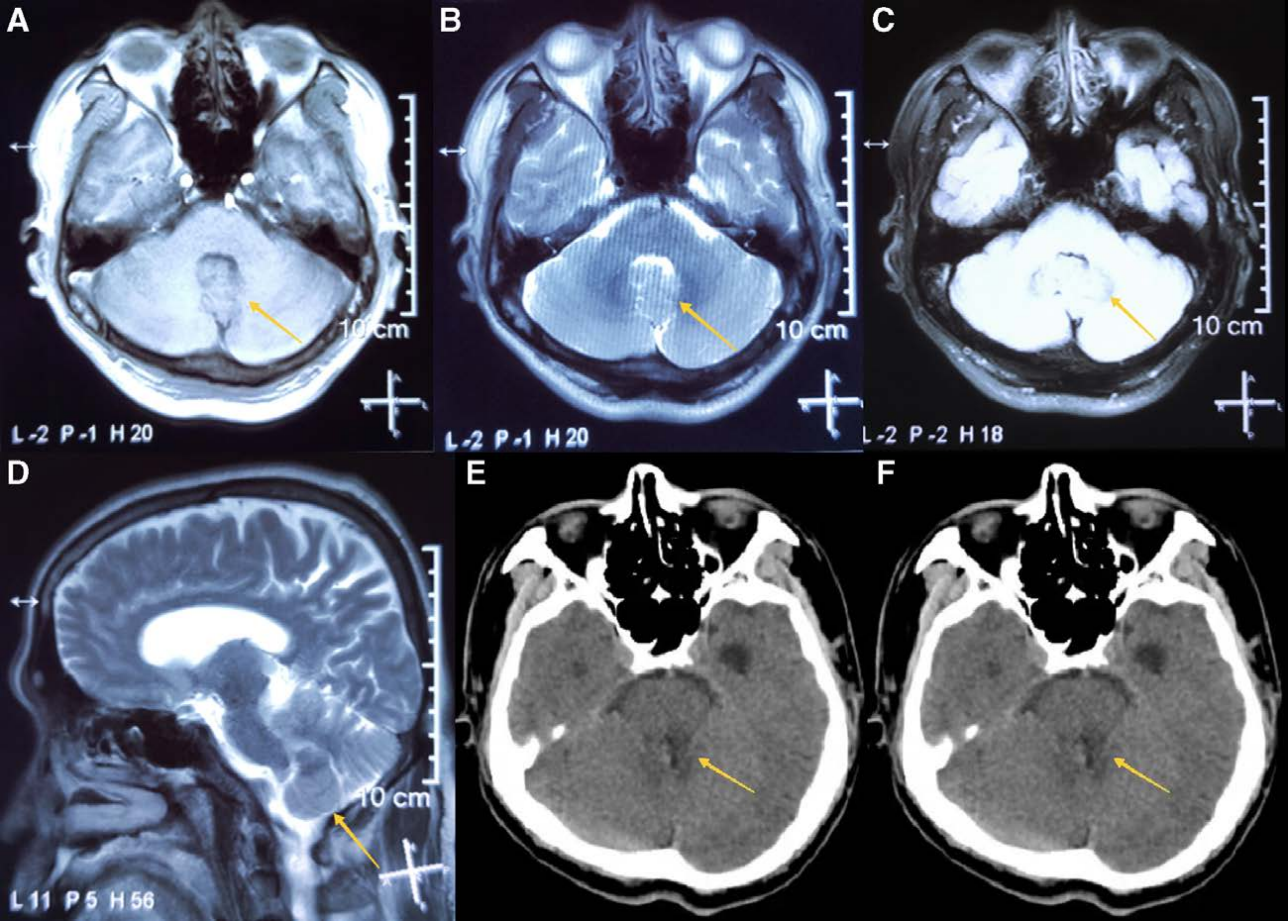
(A and B) MRI: T1-weighted and T2-weighted, images showing isointense and slightly hyperintense signals. (C and D) MRI: no obvious enhancement of the fourth ventricle lymphoma in the axial and coronal gadolinium-enhanced images. (E and F) CT: postoperative images (3 d). CT = computed tomography, MRI = magnetic resonance imaging.

Magnetic resonance imaging (MRI) (Fig. 1) demonstrated a round-shaped mass approximately 2.5 × 2.0 × 1.8 cm^3^ in size located in fourth ventricle, with slight increase T1WI signal, uniform in T2WI signal, and no enhancement following gadolinium injection. The mass lesion was homogeneously enhanced and lack of necrosis or hemorrhage. Neither cystic appearance nor calcifications was found. An additional work-up with HIV testing showed negative results.

### 2.2. Operation and histopathology

According to the radiologic features, we hypothesized that it was likely to be ependymoma. The patient underwent the operation of 4 ventricle mass resection 3 days after admitted.

A midline posterior fossa craniotomy was performed and the tumor was recessed through transvermian approach. The tumor was moderately soft, gray, translucent, with moderate blood supply. We used pathological forceps to take a small piece of tissue for fast pathological examination, and the rest of the tumor was completely removed. Histopathology revealed the lesion be a diffuse large B-cell lymphoma.

**Figure 2.**
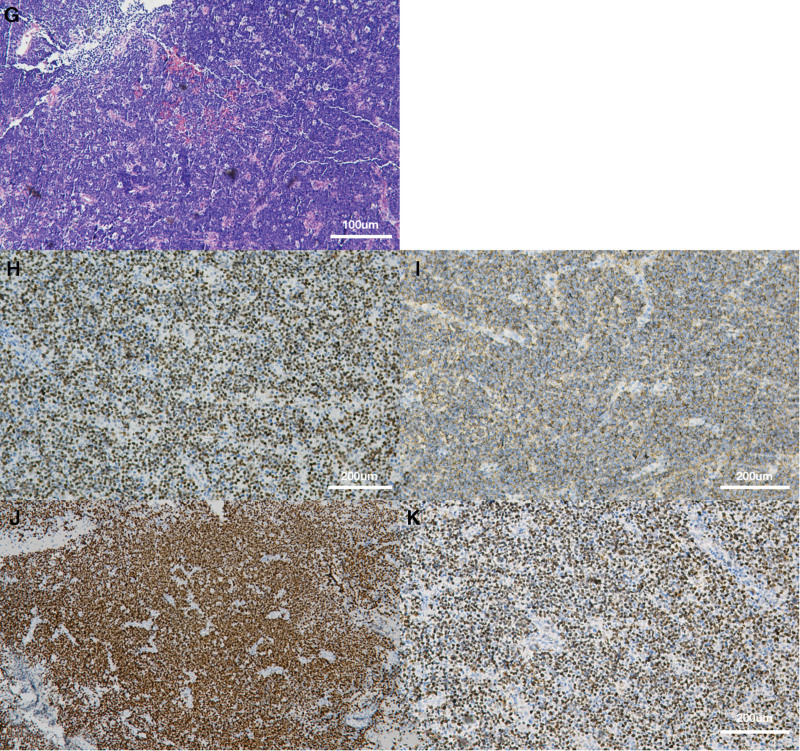
(G) Hematoxylin-eosin staining indicated that the tumor had high cell density with clumps and several pigmented deposits (100×). (H–K) Tumor cells were positive for CD10 (200×), CD20 (200×), PAX-5 (200×), and BCl-6 (200×).

Immunohistochemistry (Fig. 2) stains showed that the cells were positive for CD10, CD20, CD3, CD5, MUM1, Bcl-2, Bcl-6, and Pax-5. The proliferation index as assessed by Ki-67-staining was >90%. According to pathological result, the final diagnosis was primary central nervous system diffuse large B-cell lymphoma in the fourth ventricle.

### 2.3. Postoperative course and follow-up

Postoperatively, the patient vision loss, dizziness, and unsteady walking had relieved significantly. The patient developed fever after operation, evidence of lumbar puncture showed that the cerebrospinal fluid (CSF) was yellow in color with high protein and cell count. Then patient received lumbar drainage for a week. Finally, computed tomography (Fig. 1) suggested hydrocephalus had markedly improved, and patient discharged smoothly.

The patient received 6 cycles of intravenous and intrathecal high-dose methotrexate in the hematology department of local hospital. Along with oral zanubrutinib and lenalidomide. The patient underwent regular follow-up MRI, and no tumor recurrence was found.

## 3. Discussion

Primary CNS lymphoma itself is rare, accounting for approximately 1% of intracranial tumors. Occurs in the fourth ventricle site is more rare. The origin of such a primary tumor is unclear. Significant risk factor is acquired or congenital deficiency of immune system.^[1]^

Some studies suggest that the incidence of central system lymphomas in people without immunodeficiency is increasing, which may be related to population aging. Although the incidence of lymphoma of the central nervous system is not high, it has been increasing year by year.^[1]^Therefore, it is an important consideration in the differential diagnosis of the fourth ventricle mass lesions.^[4]^We have compiled some relevant literature and summarized some characteristics of the clinical diagnosis of lymphoma.

Clinical features: Hochberg and Miuer divided the main clinical manifestations of primary lymphoma of the central nervous system into the following 4 groups. The first are symptoms of brain involvement, mainly including headache, blurred vision, personality changes, and psychiatric symptoms. Second, symptoms of leptomeningeal involvement, these patients will have elevated lymphocytes and proteins in their CSF. Then the symptoms of ocular are universal, such as vision loss. Finally, there are uncommon symptoms of spinal cord metastases.^[5]^

Biochemical features: Serum LDH level can also help us diagnose lymphoma and is one of the international prognostic indices for NHL. Elevated LDH often indicates that NHL has extranodal metastasis and a high degree of malignancy.^[6]^ Some researchers also explored the characteristics of CSF in patients with PCNSL. In addition to elevated lymphocytes in the CSF, they found that when the level of IL-10 in CSF reached 19.62 ng/l, the sensitivity of diagnosing PCNSL was 77.5%. When IL-10 and IL-10/IL-6 were combined for diagnosis, it was found that the sensitivity and specificity could be further improved, reaching 98.9%.^[7]^ It could be used as a biomarker for PCNSL diagnosis.

Imaging features: Common tumors of the fourth ventricle include ependymoma, medulloblastoma, choroid plexus papilloma, and so on. We should be careful to distinguish the fourth ventricle lymphoma from these tumors. Intraventricular lymphoma is rare, its MRI enhancement images often showing T1 showed equal or slightly lower signal, T2 showed equal or slightly higher signal (similar to gray matter) with cotton-like or nodular shape.^[8]^ It is usually iso-intensity on MRI plain scan with uniform signal. And it often goes along with strong peritumoral edema with uniform enhancement which presents bull eye sign. However, it should be noted that if the patient is treated with glucocorticoid before the examination. The imaging results will be affected. In some cases, the tumor may disappear after using glucocorticoid.^[9]^ The identification points of various common tumors and lymphomas of the fourth ventricle have been listed in the following table (Table 1).

**Table 1:** Summary of literature on lymphoma of the fourth ventricle.

	Medulloblastoma	Meningioma	Lymphoma	Subependymoma	Subependymal giant cell astrocytoma	Choroid plexus papilloma
Site	Posterior medullary sails	Arachnoid	Intraventricular	Subependymal	Subependymal brain parenchyma	Choroid plexus
Age	Children	Adults	Immunodeficiency people	N	N	Adults
Shape	Round clumps	Clumps with clear borders	Nodule cotton balls	Oval lobulated shape	Tuberous sclerosis	Lobulated shape
MRI or CT characteristics	CT: High-density with partial calcification	MR: Signal close to brain parenchyma	MR: Uniform reinforcement	MR: Long T1 slightly long T2 often not reinforced	CT: Calcified nodules in ependyma or brain parenchyma	Significant uniform reinforcement more obvious than lymphoma
Idiosyncratic symptoms	CSF metastases	N	Increased intracranial pressure visual field defect	N	Triad symptom: Sebaceous adenoma, epilepsy, low IQ	N

CSF = cerebrospinal fluid, CT = computed tomography, MRI = magnetic resonance imaging.

Molecular pathology features: According to the European Neuro-Oncology Association guidelines for the diagnosis of immunocompetent primary lymphomas, the immunohistochemical features of diffuse large B-cell lymphoma of the central nervous system are marked by CD10, CD19, CD20, PAX-5, BCL-6, MUM1.^[2]^

Treatment progress: Some people pointed out that the best treatment plan for primary central system lymphoma should be a combination of surgical biopsy and postoperative chemotherapy.^[10]^ Because there is no significant difference in the postoperative survival rate of patients with total resection compared people with biopsy.^[11,12]^ And someone believes that total tumor resection may cause brain damage and neurological impairment.^[13]^ We searched for the previous reports on fourth ventricle lymphoma in Pubmed and summarized the results into a table (Table 2). According to these papers, it is not difficult to find that the most patients adopt complete resection, adjuvant postoperative chemotherapy, radiotherapy and other measures to ensure the survival rate.^[14]^ The current mainstream adjuvant therapy is based on high-dose methotrexate chemotherapy and intrathecal injection, supplemented by whole brain and whole spinal cord radiotherapy. In recent years, immunotherapy for PCNSL has also progressed, such as CAR-T cell therapy.^[15]^ These measures provide a guarantee for improving the postoperative survival rate of PCNSL patients.

**Table 2:** Summary of cases of primary central nervous system lymphoma of fourth ventricle since.

Publication	Age/sex	SD (mo)	Site	Relative position	MRI manifestation	Size	Surgical approach	RD	Adjuvant therapy	Follow-up (mo)	Prognosis
HaoLiu et al, 2016^[16]^	6/M	Headache	Fourth ventricle	Hypo-T1 Hper-T2	N	Posterior Fossa	TR	Chemotherapy	6	Alive	Headache
Huang et al, 2015^[17]^	61/M	3	Headache dizziness	Fourth ventricle	Hypo-T1 Hper-T2	N	Suboccipital craniotomy	TR	Chemotherapy	6	Alive
Michaela et al, 2015^[5]^	65/M	1	Weight-loss headaches blurred vision asthenia	Fourth ventricle Corpus callosum Thalamus	Hypo-T1 Hypo-T2	N	Stereotactic biopsy	PR	Chemotherapy Immunotherapy	8	Alive
YuZhu et al, 2015	66/M	2	Dizziness diplopia	Right lateral ventricle Fourth ventricle	Hypo or intense-T1 Hper or intense-T2	N	Stereotactic biopsy	PR	Chemotherapy	6	Alive
Alabdulsalam^[18]^ et al, 2014	18/M	1	Double vision facial asymmetry, tinnitus dysphagia	Fourth ventricle Third ventricle	Hypo-T1 Hypo-T2	3 × 3 × 3.5	Suboccipital craniotomy	STR	Chemotherapy Ommaya reservoir	18	Alive
Chih et al, 2014^[19]^	77/M	1	Unsteady gait vertigo, nausea vomiting	Fourth ventricle	Hypo or intense-T1 Hper or intense-T2	2.5 × 1.8 × 2	Posterior Fossa Craniotomy	TR	Chemotherapy	15	Alive
Andrew et al, 2014^[20]^	60/F	3	Diplopia	Fourth ventricle Cerebellum	Hypo-T1 Hypo-T2	N	Posterior Fossa Craniotomy	TR	Chemotherapy	9	Alive
Rakan et al, 2013^[1]^	50/M	1	Hydrocephalus symptom vomiting	Fourth ventricle	Hypo-T1 Hypo-T2	2.5 × 2 × 3	Posterior Fossa Craniotomy Endoscopic third ventriculostomy	TR	Chemotherapy Radiation therapy	18	Alive
Rahat et al, 2012^[4]^	65/F	1	Vomiting headaches	Lateral ventricle Fourth ventricle	Hypo-T1 Hypo-T2	N	Stereotactic biopsy	PR	Chemotherapy	4	Alive
C S Hill et al, 2009^[21]^	69/M	2	Vomiting	Fourth ventricle	Hypo-T1 Hypo-T2	N	Suboccipital craniectomy	TR	Chemotherapy	7	Alive
C Hae et al, 2001^[22]^	33/F	1	Vertigo headaches	Fourth ventricle	Hypo-T1 Hypo-T2	N	Posterior Fossa Craniotomy	TR	Chemotherapy	N	Alive

RD = resection degree, SD = symptom duration.

Given the favorable response of CNS lymphoma to chemoradiotherapy, PCNSL is considered a nonsurgical tumor. The current broad consensus is minimally invasive biopsy for tissue diagnosis. However, survival outcomes mentioned for PCNSL have been uniformly disappointing despite recent advanced and the often initial dramatic response to chemoradiation. German researchers found that minimally invasive surgery compared with total and subtotal resections did not significantly improve patient survival. There are also reports suggested that total tumor resection may provide not only significant clinical benefit but also elimination the cell populations with drug resistance potential. It is also correlated with longer progression-free survival and overall survival. In addition, the location of the fourth ventricle is relatively independent and special, which provides a basis for reducing the spread of the tumor and preventing the compression of the tumor from causing hydrocephalus.

## 4. Conclusion

For lymphoma in the fourth ventricle, we should pay attention to the typical symptoms such as increased intracranial pressure and visual field defect before operation. Lymphoma should be included in the routine diagnosis of fourth ventricle space-occupying lesions. For the clinical diagnosis of lymphoma, symptoms, CSF biochemical indicators, and imaging features should be combined. This has a great effect on improving the diagnosis rate of intracranial lymphoma. The optimal treatment for lymphoma of the fourth ventricle, should be to remove the mass effect of the tumor completely during surgery, and combine postoperative radiotherapy and chemotherapy. We think that biopsy is not conducive to relieve compression symptoms and may lead to tumor spread by CSF. The maximizes tumor resection can reduce the chance of tumor recurrence. And exposure to tumor mass effect, reduce brainstem compression symptoms and the incidence of hydrocephalus. It is of great benefit to patient prognosis.

## Author contributions

**Conceptualization:** Jiahui Zhao, Zongze Guo.

**Data curation:** Jiahui Zhao, Peng Cheng.

**Formal analysis:** Jiahui Zhao.

**Project administration:** Cunyi Zou.

**Resources:** Zongze Guo, Weicheng Lu.

**Software:** Jiahui Zhao, Cunyi Zou.

**Writing – original draft:** Jiahui Zhao.

**Writing – review & editing:** Cunyi Zou, Peng Cheng, Weicheng Lu.
